# Intranasally applied human olfactory mucosa neural progenitor cells migrate to damaged brain regions

**DOI:** 10.2144/fsoa-2022-0012

**Published:** 2022-07-12

**Authors:** John M Kronner, Adam Folbe, Jay Meythaler, John O Nelson, Andrei Borisov, Jean D Peduzzi

**Affiliations:** 1 Department of Ophthalmology, Visual & Anatomical Sciences, Wayne State University School of Medicine, Detroit, MI 48201, USA; 2 Department of Otolaryngology, Oakland University William Beaumont School of Medicine, Rochester, MI 48309, USA; 3 Department of Physical Medicine & Rehabilitation, Wayne State University School of Medicine, Detroit, MI 48201, USA; 4 Department of Biomedical Engineering, Wayne State University School of Medicine, Detroit, MI 48201, USA

**Keywords:** brain injury, homing, intranasal delivery, olfactory mucosa, progenitor cells, stem cells

## Abstract

**Aim:**

To determine if intranasally administered olfactory mucosa progenitor cells (OMPCs) migrate to damaged areas of brain.

**Materials & methods:**

Rowett Nude (RNU) adult rats were injured using the Marmarou model then 2 weeks later received intranasally-delivered human OMPC. After 3 weeks, rats were sacrificed and brain sectioned. The mean distances from the human OMPCs to markers for degenerative neuronal cell bodies (p-c-Jun^+^), axonal swellings on damaged axons (β-APP^+^) and random points in immunostained sections were quantified. One-way ANOVA was used to analyze data.

**Results:**

The human OMPCs were seen in specific areas of the brain near degenerating cell bodies and damaged axons.

**Conclusion:**

Intranasally delivered human OMPC selectively migrate to brain injury sites suggesting a possible noninvasive stem cell delivery for brain injury.

## Plain language summary

As a first step toward helping those with brain or spinal cord injury, human stem cells from the nose were applied to the inside of the nose of brain injured rats. These stem cells migrated to specific areas of damage in the brain. Stem cells from the nose are special, in that these cells continuously divide and form nerve cells. This study may lead to an uncomplicated treatment where tissue is taken from one side of the nose and later the stem cells from the tissue are delivered to the other side of the nose.

Diffuse axonal injury (DAI) is the most common type of traumatic brain injury that affects 2.8 million people in the USA alone in 2013 [1]. It is estimated that 1.1% of the US civilian population are dealing with long-term disabilities due to head injury [2]. Stem cells hold great promise in treating brain injury [3] and other nervous system disorders [4]. The optimal stem cell type and delivery method has yet to be determined as there are many different types of stem cells and various delivery methods being investigated.

The ideal stem cell therapy for neural injuries would be using a person’s own cells with a neural fate that are obtainable and deliverable with minimally invasive techniques. It is safer to use cells that normally become neurons or glia, in that these cells are less likely to become other inappropriate cell types [5]. Although there are many readily available stem cells such as bone marrow mesenchymal cells, fat cells and umbilical cord blood cells, none of these cells are normally fated to be neural cells. Other sources of stem cells such as embryonic, fetal and induced pluripotent stem cells suffer from problems of rejection and/or tumor formation [6–8].

Progenitor cells from the olfactory mucosa (OMPCs) are easily obtainable from the upper nasal cavity clinically. Minimally or non invasive procedures can be used to obtain OMPCs from intranasal tissue without loss of olfaction [9–11]. The olfactory mucosa is capable of lifelong neurogenesis in the adult nervous system, providing a viable source of progenitor cells. OMPCs improved function and lessened several indicators of ischemic brain damage [12–14]. Olfactory mucosa contains two types of progenitor cells: basal epithelial progenitor cells and ecto-mesenchymal lamina propria progenitor cells. Both types of cells divide rapidly in culture and give rise to neurons [15]. The basal progenitor cells continuously replace the olfactory bipolar neurons and maintain telomerase activity [16]. The ecto-mesenchymal cells differ from bone marrow mesenchymal cells in their propensity to form neurons and reduce inflammation [17,18]. Based on the location of these ecto-mesenchymal cells and SOX17 expression, these ecto-mesenchymal cells may be pericytes [19].

In terms of delivery, many methods are used to deliver stem cells including direct injection, intravenous, intra-arterial, injections into the cerebrospinal fluid (CSF) and intranasal. In preclinical studies comparing the efficacy of different delivery methods, there are conflicting results [20–23]. Direct injections result in the greatest number of cells at the injury site but injections may damage normal tissue and the injury site may be an inhospitable environment due to free radicals [24]. For DAI, injections would be extremely challenging as several very specific sites such as corpus callosum, pyramidal tract and superior cerebellar peduncle are damaged [25,26]. Less invasive methods such as intravenous, intra-arterial, CSF injections and intranasal rely on the homing capacity of the stem cells. Intravenous is minimally invasive but few cells reach the injury site, as cells must pass through the lung and other organs before reaching the brain or spinal cord [27,28]. Intra-arterial is more direct but there are reports of micro-emboli formation [29,30]. CSF injections are done by minimally invasive methods and have been used in several studies. [31,32] although there is slight risk of infection clinically [33].

Accessing the CSF is a common medical procedure but an even less invasive cellular delivery method was recently described. Danielyan and colleagues found that when drops of buffer containing mesenchymal stem cells or human glioma cells are placed on the end of the rat’s nose and aspirated, the cells reach the brain within 2 h [34,35]. Intranasal allows better survival of cells than intracranial delivery [36,37]. This delivery method of stem cells is effective in animal models spinal cord injury [38], Parkinson’s disease [39], brain injury [40], Alzheimer’s [41] and multiple sclerosis [42]. An obvious advantage due to lack of invasiveness is that repeated doses can be given [43]. We hypothesized that intranasally applied OMPCs would migrate in close proximity to degenerated cell somas and axons at specific brain regions in rats with DAI. In this study, the homing capacity of human OMPCs applied intranasally was investigated quantitatively for the first time in a brain injury model of DAI.

## Materials & methods

### Cell preparation

Human olfactory mucosa tissue samples were obtained from surgical remnants (Wayne State University Institutional Review Board #052711) during skull base surgeries. Tissue was cut in small pieces using two #11 surgical blades in a small drop of Hank’s Balanced Salt Solution (HBSS, Hyclone, UT, USA) then incubated in 2 ml of dispase I (Stem Cell Technology) and 2.4 μl of DNase II (Roche) for 30 min at 37°C, pipetting up and down halfway through. Between steps, the solution was centrifuged and supernatant discarded. Cells were then incubated in 1.5 ml of Accutase (Innovative Cell Technology, CA, USA) under the same conditions. OMPCs were incubated overnight in DMEM/F12 (Gibco) with 10% serum obtained from the patient. Serum was prepared by spinning blood for 15 min at 1000 RCF then using the supernatant. To select for stem cells, the following media was used: DMEM/F12 with 2% xeno-free B-27 (Gibco), (EGF, 50 ng/ml, Peprotech, NJ, USA), basic (FGF-2, 25 ng/ml, R&D systems, MN, USA), 1% antibiotic/antimycotic (HyClone). Cells are plated in sterile non tissue culture treated flasks.

### Injury model

**Figure 1.**
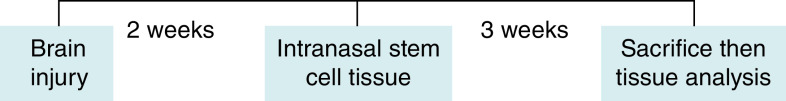
Timeline of experiments. Rats received a diffuse axonal brain injury using the Marmarou model then received intranasally applied olfactory mucosa progenitor cells 2 weeks later. At 3 weeks after olfactory mucosa progenitor cell delivery, rats were sacrificed and tissue analyzed.

All animal procedures were approved by the Wayne State University Institutional Animal Care and Use Committee. Nude RNU adult rats (Charles River, MA, USA) are athymic (immune deficient) to prevent rejection of the human OMPCs [44]. Animals (n = 6) were housed in sterile cages, received sterile food and cages changed in a laminar flow hood. The timeline of experiments is given in Figure 1.

Rats were anesthetized with 1.5–2% isoflurane with oxygen at 1 l/min using a calibrated vaporizer and a nose cone. Brain injury was done using the Marmarou Acceleration Impact model of DAI [45,46] that consists of dropping a 450 g brass weight from a height of 2 m that impacts a metal disc cemented to the rat’s skull in the midline between bregma and lambda. The weight is dropped in a 3 m long hollow plexiglass cylinder with an outer diameter 25 mm attached by supports bolted to the wall for stability. Some prefer to call this injury ‘traumatic axonal injury’ [47]. Briefly, after the scalp is shaved and cleaned 3x with betadine then 70% alcohol, a midsagittal scalp incision (2 cm) was made and the underlying muscles and connective tissue retracted laterally. Cranioplastic cement was used to attach a round stainless-steel disc helmet (10 × 3 mm) directly to the skull. Anesthesia is briefly discontinued and the animal placed in a prone position on a 12 × 12 × 43 cm polyurethane foam (Foam to Size Inc., VA, USA) in a plexiglass box. Details on obtaining consistent results with the foam are reported [48]. The device tube was then positioned directly above the disc. The injury was delivered by dropping the weight suspended by a fishing line. The box was moved immediately after impact to avoid a secondary injury. The rats were placed back on anesthesia and the helmet and cement were removed and the skin closed with 4–0 nylon suture. OMPCs were applied intranasally 2 weeks after injury.

### Stem cell administration

One day prior to stem cell administration, cells were incubated overnight in a combination of stem cell media and DAPI (20 mg/ml, Abcam). DAPI was effective as a secondary method to identify stem cells as was done in a previous study [49]. The next morning, cells were counted using a hemocytometer then centrifuged for 3 min at 200 rpm. Supernatant was removed and cells were diluted to a concentration of 1.2 million cells per 24 μl of HBSS. For intranasal administration, a syringe with BD Intramedic™ PE 10 tubing (25 mm length marked as determined to reach the olfactory mucosa) was used. Rats were anesthetized with isoflurane and received 6 μl hyaluronidase (100 U, Sigma) in each nostril two-times, for a total administration of 24 μl [35]. An hour later, the cells diluted in HBSS or HBSS alone were administered intranasally just as the hyaluronidase was delivered. Rats were randomly assigned to receive OMPC or HBSS. There was a total administration of 1.2 million OMPCs in 24 μl for each rat that received stem cells.

### Immunofluorescence

About 3 weeks after the cell administration, rats were terminally anesthetized with an intraperitoneal injection of pentobarbital 125 mg/kg and perfused through the left ventricle with saline followed by 10% formalin, post fixation for 24 h then equilibrated with 30% sucrose. Rat brains were cut using a horizontal sledge type microtome at a thickness of 30 microns and sections placed in cryoprotectant for long-term storage in the freezer [50]. The brains were cut at a coronal orientation until the cerebellum was reached. For the cerebellum and brain stem, the brain was sectioned in parasagittal plane. Twenty random tissue sections were mounted on gelatin coated slides. Slides were rinsed in PBS for 2 minutes, three-times. Slides were placed in 100°C citrate buffer in a preheated 100°C oven for 1 hour for antigen retrieval. Slides were rinsed in 1X phosphate-buffered saline (PBS) for 2 min, three-times. Sections were blocked using in 5% normal goat serum (NGS), 1% bovine serum albumin (BSA), 0.3% triton-X in PBS for 90 min. Primary antibodies were diluted in 2% NGS, 1% BSA in PBS and incubated overnight at 4°C. As a control, primary antibody was omitted to detect any non specific immunostaining. The following antibodies were used: mouse anti human nuclei (Millipore) 1:100, mouse anti-β-APP (Invitrogen, axonal swellings [51]), rabbit anti-phospho-c-jun 1:250 (Cell Signaling, p-c-jun, cell soma atrophy [52]) and rabbit anti-β-tubulin III (Millipore, neuronal marker [53]). Slides were rinsed in PBS for 2 min, three-times. Secondary antibodies used were Alexa fluor 488 goat anti mouse (1:250) and Alexa fluor 546 anti rabbit (1:200) that were diluted in 2% NGS, 1% BSA in PBS and incubated for 2 h in the dark. Slides were rinsed in 1x PBS for 2 min, three-times then coverslipped using 50:50 glycerol: PBS.

### Data analysis

The distribution of fluorescent cells was photographed using a Nikon Eclipse 90i fluorescent microscope that automatically photographed the entire section using multiple wavelengths. The mean distances from the OMPCs to the following sites were compared using Neurolucida (BMF Bioscience, Williston, VT, USA) : axonal injury (β-APP); cell soma atrophy (p-c-jun); random site (randomly generated X, Y coordinate for each tissue section). These measurements were used to determine if the OMPCs were more likely to migrate to injury sites than other random sites in the rat brain. The distances were compared using one-way analysis of variance (ANOVA) followed by the Tukey–Kramer *post hoc* test. Significance was set at p < 0.05.

## Results

All rats survived the brain injury and OMPCs were applied 2 weeks after injury using tubing inserted into the nasal cavity of the rats. Rats were sacrificed at 3 weeks after intranasal application. No DAPI or anti human immunolabeling was present in the sections from rats that received HBSS alone. In the rats that received human OMPC, cells with anti human nuclei immunostaining were visible. Double immunofluorescent revealed that the DAPI-labeled cells were also immunoreactive for anti human nuclei and β-tubulin III.

**Figure 2.**
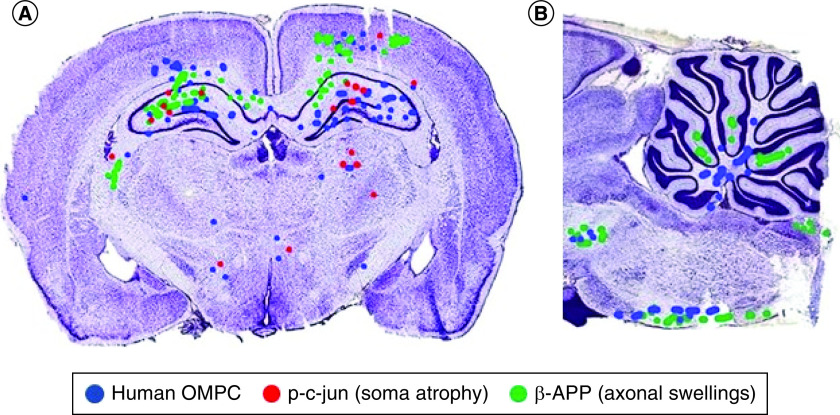
Distribution of intranasally delivered olfactory mucosa progenitor cells to axonal damage and cell atrophy. Mapping of OMPC cells and p-c-jun^+^ and β-APP^+^ profiles in a coronal **(A)** and parasagittal section **(B)** OMPCs were found in injury sites. Injured regions include the cerebral cortex, cerebellum, cerebral peduncles and brain stem tracts. Some of the dots do not appear perfectly circular because of the overlap of dots. In most brains, the number of human OMPCs on each side differed by less than 11%. In this illustrated section, there is more than 11% difference in the number of OMPCs between the two sides and higher indicators of damage (β-APP and p-c-jun) on the side with more OMPCs. A limitation of this study is that labeled cells were not photographed using a confocal microscope to visualize processes of the OMPCs. OMPC: Olfactory mucosa progenitor cell.

After intranasal application, OMPCs migrated to regions of cell soma atrophy and axonal swellings or end bulbs (Figure 2). Long axonal tracts such as the pyramidal tract, medial lemniscus and superior cerebellar peduncle had numerous labeled OMPCs. Cell soma atrophy as detected by p-c-jun was most prominent in the hippocampus particularly CA1. Axonal swellings and end bulbs as revealed by β-APP^+^ were present in the corpus callosum, deep layers of the cerebral cortex, CA2 of the hippocampus, fasciculus cuneatus and grey matter of the cerebellum. In the cerebellum, the OMPCs were in the white matter while the was little overlap with the β-APP^+^ profiles. In most brains, the number of human OMPCs on each side differed by less than 11%. In one brain, there were more profiles positive for β-APP and p-c-jun that are indicators of damage. On that side, there were more OMPCs as indicated by anti human antibody and DAPI (Figure 2).

By photographing the entire sections at multiple wavelengths, it was possible to map the position of the human nuclei, DAPI, p-c-jun^+^ and β-APP^+^ cells. The mean distance (+SD) from OMPC to regions of axonal damage (β-APP^+^) was 71.8+60.3 μm; OMPC to degenerating cell bodies (p-c-jun^+^) was 188.7 + 320 μm; OMPC to a random site 3929.3 + 2636.3 μm. There was a significant difference at the p < 0.05 level for the three distances using a one-way ANOVA (F[2429] = 224.99; p = 1.51E^-67^). *Post hoc* comparisons using the Tukey–Kramer indicated that the distance from OMPC cells to β-APP^+^ or p-c-jun^+ ^was different than OMPC cells to a random site.

## Discussion

In our study, the homing capacity of human OMPC applied intranasally was determined quantitatively for the first time in a brain injury model of DAI.

Human OMPC migrated into the brain after delivering the cells into the nasal cavity using a fine tube to apply the cells to the olfactory mucosa. Using tubing inserted in the rat’s nose may have allowed more cells to reach the olfactory mucosa as opposed to placing a droplet with stem cells at the end of rat’s nose. Using a droplet at the end of the nose, the greatest number of cells outside of the brain is the stomach [35]. The administered OMPCs migrated to sites of damage in brain injured rats. The human stem cells were found in close proximity to axon swellings revealed with β-APP antibody and degenerating cell bodies detected using p-c-jun antibody. The only region where the OMPCs did not appear overlap the β-APP^+^ was the cerebellum. It may be that OMPCs are still migrating at 3 weeks after delivery. Cells are also known to migrate at different rates in different areas during development [54]. Another possibility is that these OMPCs are migrating from the ventricle as opposed to the subarachnoid space that may delay migration.

In this study, we used two different methods to identify the human OMPC: anti human nuclei antibody and DAPI. Although DAPI has been used to label dead cells, the permeability to DAPI depends on the type of cells, incubation period and concentration of DAPI [55]. DAPI has also been used to label living neurons [56]. Cai and colleagues [49] found that stem cells are permeable to DAPI and used DAPI labeled mesenchymal cells to follow their cell migration for several weeks. In their study, DAPI labeled cells directly correlated to the Feridex-labeled cells.

### Mechanism of migration

There are four major pathways proposed as possible routes on how stem cells enter the brain within hours after intranasal delivery. These include the perivascular route, CSF, lymphatics and cranial nerves [35,57–59]. Several or all of these routes may be used by the stem cells: the perivascular space between blood vessels and the astrocytic end feet is believed to allow transport of fluid and other substances between the intracellular space, CSF and blood vessels. This space was named glymphatic system [60] in the belief at that time that there were no lymphatics in brain. Recently lymphatics were found in the dura of experimental animals and humans [61–63]. In addition, a recent study found lymphatic endothelial cells in the human perivascular space that also contained T cells [64]. Stem cells may travel to the brain using this route as it is known that glioma cells and immune cells migrate in this space [64,65]; the cells may directly enter the CSF as the subarachnoid space extends for a short distance around the olfactory nerve; cells may directly enter the lymphatics as there are a large number of lymphatic vessels in the olfactory mucosa. Cells may then enter the CSF from the lymphatics. There are anatomical connections between the CSF and lymphatic vessels in nasal cavity of rats and humans [66]; stem cells might follow the olfactory or trigeminal cranial nerves to enter the brain as both nerves are present in the nasal cavity [64,66]. The interplay of these pathways complicates interpretation. For example, the spaces surrounding cranial nerves contain lymphatic vessels [64].

After cells enter the brain, there are several possibilities on how the cells migrate to areas of injury. The perivascular space may form the pathway to reach these areas. It is interesting to note that OMPCs are found not only in the subarachnoid space but also in the ventricle as also noted by others [34]. One possibility is that the cells enter the olfactory bulb and follow the rostral migratory stream to reach the ventricle [67] as it is thought to be a bidirectional pathway [67]. Our results demonstrate that the OMPCs migrate to close proximity to the axonal swellings and degenerating cell bodies.

### Mechanism of homing

The attraction of stem cells to sites of damage has been explored in several studies [68–70]. Important roles for two chemokines are apparent: stromal derived factor-1 (SDF1, also called CXCL12) and monocytic chemoattraction protein (MCP, also called CCL2). When CXCL chemokine receptor 4 (CXCR, receptor for SDF) is blocked using monoclonal antibody *in vitro* [68], migration of human neural stem cells to areas of damage in organotypic cortical explants is severely reduced. When SDF-1 is blocked with antibody in a rat hypoxic-ischemic model, the migration of human umbilical cord blood cells is curtailed [69]. Similar results are obtained with MCP or its receptor CCR2. Mice lacking MCP or CCR2 exhibit significant decreases in the number of migrating neuroblasts to the ischemic striatum [70].

Previous studies have already shown that administration of hyaluronidase to rats before intranasal delivery of cells, allows for the cells to migrate to the brain more effectively due to the breakdown of hyaluronic acid which loosens the barrier of the nasopharyngeal mucosa [35]. We utilized this method for all of our intranasal administration of OMPCs to promote cell migration. After intranasal delivery of OMPCs we found that they were able to migrate to sites of injury. These injury sites include: cerebral cortex, cerebellum, cerebral peduncles and brain stem tracts as evident in previous studies using this injury model [71]. The cells were also found in the subarachnoid space and ventricles suggesting migration of the cells through the CSF. The labeling of OMPCs using anti human nuclei antibody was confirmed by the DAPI staining. Due to the rats being sacrificed three weeks after cell administration, some of the cells had faded DAPI staining.

If the weight drop impacts on one side of the brain more than the other, there is evidence of more injury sites on that side of the brain. Due to this inconsistency we were actually able to learn more about the intranasal delivery of the OMPCs compared with those rats with equal axonal injury on both sides of the brain. We were able to observe that the rats with greater injury on one side of the brain had a greater concentration of OMPCs on that side of the brain than on the other side suggesting injury specific attraction (Figure 2).

### Delivery method effectiveness

Administering the cells through the rat’s nose was a simple and effective method in delivering OMPCs to head injured rats. Using tubing inserted into the rat’s nose at a specified depth, we were able to administer cells closer to the olfactory mucosa, which allowed for a greater retention of cells than having the rats snort the cell suspension into the nose. This method is less invasive than administering the cells directly into the CSF.

Not only do the OMPCs migrate to sites of injury, but there was also evidence of the cells differentiating into neurons. Through the utilization of antibodies against β-Tubulin III, we were able to determine that the OMPCs could potentially be used to fill the void of neurons that had been damaged due to DAI as a study has demonstrated that transplanted OMPCs receive synapses from and synapse upon endogenous cells after spinal cord injury [72].

## Clinical translation

A future clinical protocol would entail removing half of the olfactory tissue from one side of the nose [11] or obtaining OMPCs using a brush inside the nasal cavity [8]. Either method could be done without any permanent damage to olfaction [9]. The OMPCs can be expanded in culture, defined and tested before administering to the opposite side of the nose intranasally. The OMPCs can be obtained and delivered in the same person that avoids the problem of rejection and ethical concerns. Safety profile is further increased because the cells are taken and applied to the same area of the body. This procedure would not only be applicable for patients with mild to moderate brain injuries, but also patients in the intensive care unit due to the low risk and minimal invasiveness. OMPCs have the fastest rate of neurogenesis in the adult brain and their normal fate is neurons. OMPCs may be safer than other stem cells that normally develop into cartilage and bone.

## Conclusion

OMPCs were found in the subarachnoid space and ventricles of the brain confirming one method of cell migration through the CSF after intranasal delivery. In this study, quantitative analyses revealed that OMPCs delivered intranasally specifically target sites of axonal injury and cell soma atrophy in rats with a brain injury.

**Summary points**

- The olfactory stem cells divide continuously and their normal fate is neurons so are an ideal source of neural stem cells.
- Human olfactory stem cells delivered intranasally migrate to specific areas of brain damage in rats.
- Olfactory stem cells can be obtained and delivered non-invasively so may represent a promising treatment for neural injuries.
